# The nuclear lamina during human spermiogenesis

**DOI:** 10.1186/1750-1172-10-S2-O22

**Published:** 2015-11-11

**Authors:** Michael Mitchell

**Affiliations:** 1 Aix Marseille Université, INSERM, GMGF UMR_S 910 13385, Marseille, France

## 

The nuclear lamina takes centre stage during spermiogenesis, the post-meiotic phase of spermatogenesis, when haploid round spermatids differentiate into spermatozoa: the acrosome and flagellum develop at opposite nuclear poles, the nucleus elongates and, as the nuclear histones are replaced with protamines, the chromatin condenses, to produce the highly compacted pyriform nucleus of the mature spermatozoa. In rodent spermatids, the nuclear lamina contains lamin B1 and lamin B3 a specific isoform of lamin B2 with a shortened rod domain, and A-type lamins are absent[1,2], but nothing is known about the structure of the nuclear lamina during human spermiogenesis. We are studying the nuclear lamina during human spermiogenesis. We have shown that the human nuclear lamina contains lamin B1 and, distinct from rodents, lamin B2. We also described a transcript potentially encoding a human lamin B3 that, like its mouse counterpart[3], induces severe nuclear deformation when expressed in HeLa cells[4]. In human, lamin B1 and B2 localise to the nuclear periphery in spermatids except in the region covered by the acrosome. They are seen to recede to the posterior pole of the nucleus as the spermatids progress through spermiogenesis. Lamin B1 was observed on 30-40% of ejaculated spermatozoa, while lamin B2 was not detected. The percentage of B1-labelled spermatozoa dropped at least 6-fold when spermatozoa with normal head density were selected, indicating that lamin B1 labels immature spermatozoa lacking a fully compacted nucleus, and may therefore be a marker of poor sperm quality. The comparison of the human nuclear lamina with that of the mouse suggests that lamin B1 and B3 have critical roles during mammalian spermiogenesis.
